# Antifungal and Phytotoxic Activities of Isolated Compounds from *Helietta parvifolia* Stems

**DOI:** 10.3390/molecules28237930

**Published:** 2023-12-04

**Authors:** Victor Pena Ribeiro, Joanna Bajsa-Hirschel, Prabin Tamang, Kumudini Meepagala, Stephen O. Duke

**Affiliations:** 1 Agricultural Research Service, Natural Products Utilization Research Unit, U.S. Department of Agriculture, University, MS 38677, USA; vpribeir@olemiss.edu (V.P.R.); joanna.bajsa-hirsche@usda.gov (J.B.-H.); prabin.tamang@usda.gov (P.T.); 2 National Center for Natural Products Research, University, MS 38677, USA; sduke@olemiss.edu

**Keywords:** *Helietta parvifolia*, herbicidal, fungicidal

## Abstract

The identification of natural and environmentally friendly pesticides is a key area of interest for the agrochemical industry, with many potentially active compounds being sourced from numerous plant species. In this study, we report the bioassay-guided isolation and identification of phytotoxic and antifungal compounds from the ethyl acetate extract of *Helietta parvifolia* stems. We identified eight compounds, consisting of two coumarins and six alkaloids. Among these, a new alkaloid, 2-hydroxy-3,6,7-trimethoxyquinoline-4-carbaldehyde (**6**), was elucidated, along with seven known compounds. The phytotoxicity of purified compounds was evaluated, and chalepin (**4**) was active against *Agrostis stolonifera* at 1 mM with 50% inhibition of seed germination and it reduced *Lemna pausicotata* (duckweed) growth by 50% (IC_50_) at 168 μM. Additionally, we evaluated the antifungal activity against the fungal plant pathogen *Colletotrichum fragariae* using a thin-layer chromatography bioautography assay, which revealed that three isolated furoquinoline alkaloids (flindersiamine (**3**), kokusagenine (**7**), and maculine (**8**)) among the isolated compounds had the strongest inhibitory effects on the growth of *C. fragariae* at all tested concentrations. Our results indicate that these active natural compounds, i.e., (**3**), (**4**), (**7**), and (**8**), could be scaffolds for the production of more active pesticides with better physicochemical properties.

## 1. Introduction

In modern agriculture, weeds and microbes cause significant economic damage by reducing crop yield in both quality and quantity. To combat this challenge, the commercial agriculture sector mainly relies on the application of chemical herbicides and fungicides to mitigate these problems primarily due to their high level of efficacy, affordability, ease of application, and widespread availability [1]. However, the widespread use of these chemicals has led to problems such as the emergence of herbicide and fungicide resistance [2,3,4,5]. Genetically modified herbicide-resistant crops, particularly glyphosate-resistant (GR) crops, were introduced in 1996 [6], making reliance on herbicides for weed management even more intense. The initial detection of GR ryegrass (*Lolium rigidum*) in Australia in 1996 posed an unexpected challenge for weed scientists [5,7]. There has been a sharp rise in the prevalence of GR weeds since GR crops were introduced [5,8]. The synthetic herbicides available to farmers represent only a few modes of action (MOA) [9]. According to the Herbicide Resistance Action Committee (HRAC), there are 25 modes of action (MoA) for herbicides. No significant new herbicide MOA has been introduced in a commercial herbicide for more than 40 years [10]. Evolution of resistance to most of these MOAs is well documented [3,5,9]. Weed resistance is so intense in some places that farmers are running out of effective chemical weed management options. Thus, the search for herbicides with new MOAs is currently intense, and natural products are a potential source for such herbicidal molecules due to the evolution of natural defense mechanisms via the production of biologically active secondary metabolites, which help organisms survive in their respective ecological niches.

Similarly, the excessive and indiscriminate use of fungicides has led to adverse effects on humans, animals, and the environment [2,4]. These negative consequences are further exacerbated by the evolution and spread of fungal strains that have evolved resistance to fungicides [11]. The repetitive application of single-site MOA fungicides is one of the contributing factors in the increased development of fungicide resistance [12]. This overreliance on a limited set of fungicide MOAs can lead to the selection of fungal populations with reduced sensitivity to these chemicals, making disease control more challenging and underscoring the importance of implementing more sustainable and diversified strategies for managing fungal pathogens in agriculture.

Natural product-based pesticides, including herbicides and fungicides, have gained broad public acceptance due to their perceived toxicological and environmental safety compared to synthetic pesticides. One of their advantages is the relatively shorter environmental half-lives compared to most synthetic pesticides, which reduces their potential negative impact in the ecosystem and on human health. Moreover, natural products exhibit a wide range of chemical structures with evolved biological activity, allowing for the potential discovery of novel and multiple MOAs [13,14,15]. Multiple MOAs can be advantageous for weed and disease management, as this reduces the likelihood of resistance development in the pests. Thus, natural products continue to be investigated for novel compounds with new MOAs that can be used as pesticides that function against resistant weeds and fungi [16,17,18]. 

*Helietta parvifolia*, commonly known as Baretta, is a tree that belongs to the Rutaceae family. This plant family is renowned for producing a diverse range of chemical constituents, many of which exhibit a wide range of biological activities, including neurodepressant effects [19] and antimicrobial [20] and antiparasitic properties [21]. *H. parvifolia* is a perennial flowering tree native to the Lower Rio Grande Valley of Texas and northeastern Mexico. Its wood is primarily utilized in construction for building houses and fences, and it is also a popular choice for erosion control due to its extensive root structure [22]. From a chemical perspective, *H. parvifolia* is rich in quinoline alkaloids and possesses various beneficial properties such as anti-inflammatory properties [23], fungicidal and insecticidal activities [24], and anticholinesterase activity [25]. With this knowledge, we investigated *H. parvifolia* as a source of active compounds for natural product-based fungicides and herbicides. Our study focused on the ethyl acetate extract derived from the stems of *H. parvifolia*, as it was the most biologically active fraction when tested using bioautography for antifungal activity against *C. fragariae*.

## 2. Results and Discussion

**Figure 1:**
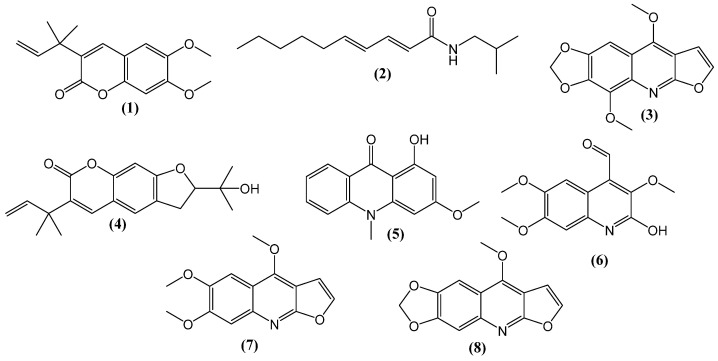
Chemical structures of compounds purified from the stem ethyl acetate extract of *Helietta parvifolia*: rutacultin (**1**), pellitorine (**2**), flindersiamine (**3**), chalepin (**4**) 1-hydroxy-3-methoxy-10-methylacridone (**5**), 2-hydroxy-3,6,7-trimethoxyquinoline-4-carbaldehyde (**6**), kokusagenine (**7**), and maculine (**8**).

Eight compounds were isolated from the ethyl acetate fraction of the *H. parvifolia* stem. These included two coumarins and six alkaloids, identified as rutacultin (**1**) pellitorine (**2**), flindersiamine (**3**), chalepin (**4**), 1-hydroxy-3-methoxy-10-methylacridone (**5**), 2-hydroxy-3,6,7-trimethoxyquinoline-4-carbaldehyde (**6**), kokusagenine (**7**), and maculine (**8**) (Figure 1). While the furoquinoline alkaloids flindersiamine (**3**) and kokusagenine (**7**) had been previously identified in the leaves of *H. parvifolia* [25], the other compounds isolated and identified from the stem of *H. parvifolia* had not been previously reported from this species.

**Figure 2:**
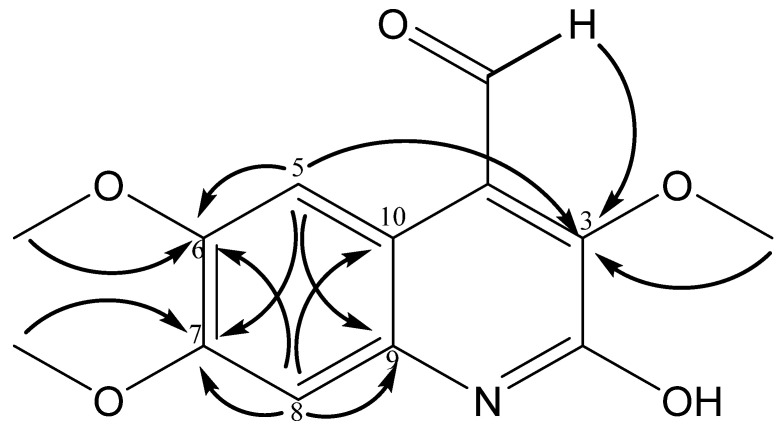
HMBC correlations for 2-hydroxy-3,6,7-trimethoxyquinoline-4-carbaldehyde.

Compound **6** was identified as a new quinoline alkaloid. The ^1^H (400 MHz) NMR spectrum revealed a singlet at *δ* 10.22 characteristic of an aldehyde. Additionally, the ^1^H spectrum also revealed the presence of three methoxyl groups. ^13^C and DEPT-135 spectra showed the presence of three OCH_3_ groups, the absence of CH_2_ groups, seven quaternary carbons, and three CH groups including the carbonyl group at 189.9 ppm. The resonance at 164.05 ppm was assigned to the hydroxylated carbon of the quinolinic ring based on HMBC correlations. HMBC correlations of the proton at *δ* 7.25 were observed with carbons at 168.3 (C-3), 155.3 (C-6), 145.8 (C-7), and 137.8 (C-9) ppm, confirming that the aromatic proton is at C-5, whereas the proton at δ 6.85 showed correlations with 155.3 (C-6), 145.8 (C-7), 137.8 (C-9), and 108.9 (C-10) ppm, confirming that the other aromatic proton is at C-8 (Figure 2) (see Supplementary Material Figure S4). The ^1^H and HSQC spectra indicated two aromatic protons at *δ* 7.25 and 6.85. The HR-DART positive mode mass spectrometric analysis exhibited the molecular ion [M + H]^+^ at *m*/*z* 264.0892, corresponding to the molecular formula C_13_H_14_NO_5_ (see Supplementary Material, Figure S1). The analysis of NMR and mass spectra suggests the structure of compound **6** to be as shown (Figure 2).

All the isolated compounds were assessed for phytotoxicity against lettuce and bentgrass at 1 mM. This bioassay evaluates the phytotoxicity of compounds against dicotyledonous (lettuce) and monocotyledonous (bentgrass) plants. At this concentration, most of the compounds had phytotoxic effects on bentgrass, while only compound **1** had a mild effect on lettuce. Notably, compounds **4** and **6** showed the most potent activity against bentgrass, with a ranking of 3, indicating 50% inhibition of seed germination. Compounds **2** and **3** exhibited moderate activity (a ranking of 2) against bentgrass (Table 1). None of these compounds were as active as the synthetic herbicide atrazine.

**Figure 3:**
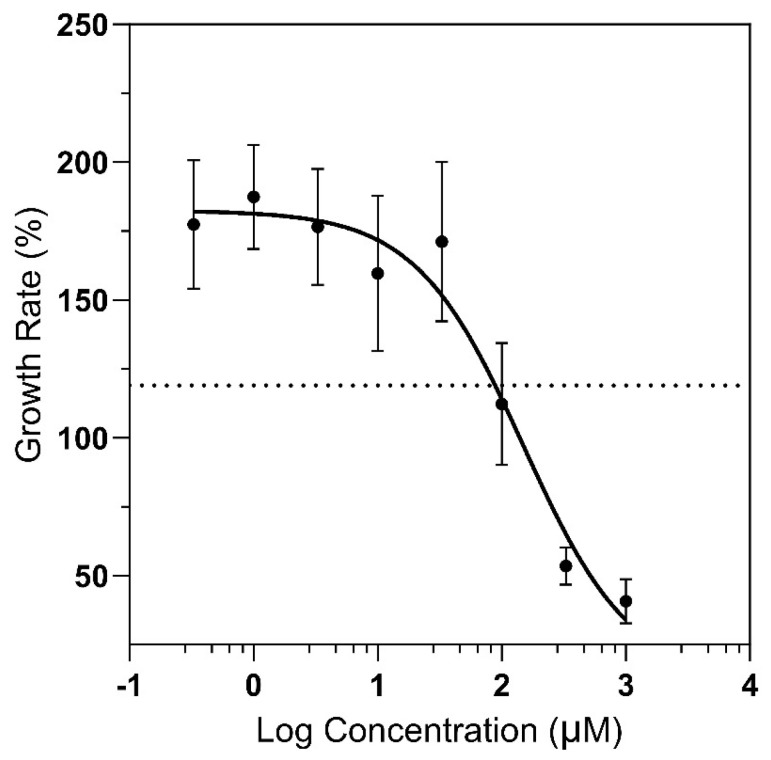
Effects of chalepin (**4**) on the growth (% of initial frond area) of *L. pausicostata* at varying concentrations after 7 days of exposure. Each treatment was carried out in triplicate. The dotted line is 50% of the control. Error bars are ±1 standard error of the mean.

The two most phytotoxic compounds against bentgrass were chosen for further evaluation in dose−response bioassays using duckweed (Figure 3). Compound **4** (chalepin) reduced growth by 50% at 168 μM, whereas compound **6** had an IC_50_ value of >1000 μM. The phytotoxicity exhibited by chalepin (**4**) was found to be more potent than that reported for certain synthetic herbicides, such as glyphosate and asulam, with IC_50_ values of 388 μM and 407 μM, respectively, under comparable experimental conditions [26].

Chalepin has been reported to inhibit the growth of excised wheat coleoptiles at concentrations as low as 10 µM [27]. It is impossible to compare these results with ours, as the coleoptile is not a photosynthetic tissue, and the part to the coleoptile used in this bioassay contains no meristem. For these reasons, no companies conducting herbicide discovery research use the coleoptile for the detection of phytotoxicity; they use whole plant bioassays (e.g., seed and seedling-based) and, in some cases, duckweed (*Lemna* spp.) e.g., [28], as we did in this paper. In the same study [27], chalepin had no significant effect on the growth of lettuce roots at concentrations up to 1 mM, but inhibited shoot growth at concentrations as low as 10 µM. Chalepin inhibited both root and shoot growth of tomato (*Lycopersicon esculentum*) as low as 10 and 30 µM, respectively, and the growth of both root and shoots of onion (*Allium cepa*), both at 30 µM. Anaya et al. [24] reported chalepin to inhibit the growth of roots of *Amaranthus hypochondriacus* and *Echinocloa crus-galli*. These authors considered it to be an allelochemical involved in plant–plant interactions, although no rigorous proof of this chemical ecology role was provided. Mammalian toxicity [29] might preclude chalepin from use as a natural product pesticide, although it has been reported to have therapeutic potential in treating human diseases [30]. These authors point out that this molecule fits Lipinski’s rules [31] for the physicochemical properties of most pharmaceuticals. The properties of Lipinski for effective pharmaceuticals are very close to those for good pesticides [32]. 

**Figure 4:**
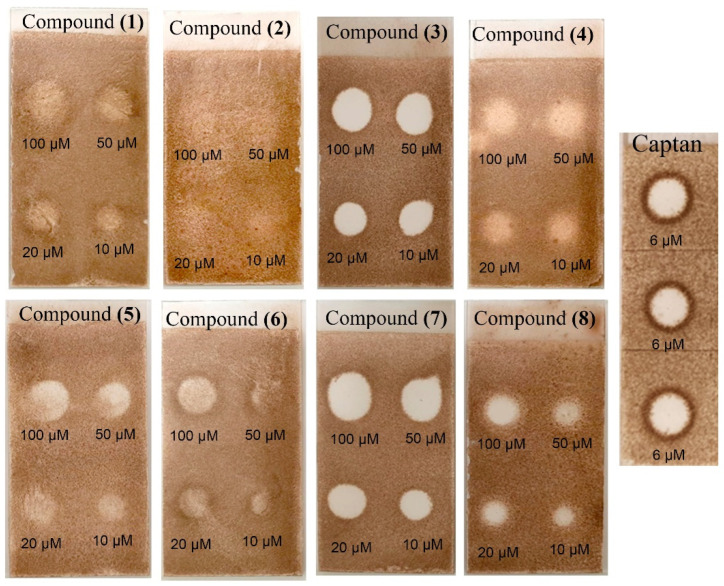
TLC bioautography of isolated compounds against *Colletotrichum fragariae*.

The antifungal properties of the isolated compounds were additionally assessed against the plant pathogenic fungus *Colletotrichum fragariae* using a thin-layer chromatography bioassay. Among them, weak antifungal activity was observed for compounds **1**, **2**, **4**, **5**, and **6**. However, compounds **3**, **7**, and **8** displayed a good antifungal activity (Figure 4). Both compound **3** and **7** exhibited the strongest inhibitory effects on the growth of *C. fragariae* at all tested concentrations. In contrast, compound **8** only inhibited the growth of *C. fragariae* well at a concentration of 100 μM. The positive control (captan) was more active than any of the tested natural compounds.

Certain alkaloids possess antifungal properties that are relevant to both human and plant crop pathogens [33]. Some alkaloids found in *H. parvifolia* have inhibitory activity against other fungal strains. The alkaloids maculine (**8**), flindersiamine (**3**), and kokusagenine (**7**), which were previously isolated from the bark of *Helietta apiculate*, have inhibitory activity against the fungus *Candida krusei* [34]. These furoquinoline alkaloids were also isolated from *Raualinoa echinata* and inhibited the growth of *Leucoagaricus gongylophorus*, a fungal plant pathogen [35]. Pellitorine (**2**) was reported to be an inhibitor to several fungi of medicinal interest (*Aspergillus flavus*, *A. fumigates*, *Coniophora puteana*, *Fibrophoria vaillentii*, *Fusarium proliforatrum*, and *Rhizopus* SP) but without activity on several others (e.g., *A. niger*, *Fusarium oxysporum*, and *Sclerotium rolfsii*) [36]. Biavatti et al. found flindersiamine (**3**), kokusagenine (**7**), and maculine (**8**) to inhibit the growth of the fungus *Leucoagaricus gongylophoorus* by 50 to 100% at 100 µg mL^−1^ [37]. 

Furoquinoline alkaloids exhibit a broad spectrum of biological characteristics. Our study highlights *H. parvifolia* as a source of furoquinoline alkaloids, with the potential for application as natural pesticides.

Even though pellitorine (**2**) had low herbicidal and fungicidal activity in our assay, we note that this compound has been reported to have sufficient activity against insects to be considered a lead for the development of new insecticides [38,39,40]. Indeed, more active and safer pesticides might be chemically derived from the compounds described here. For example, Valdez et al. found that certain derivatives of kokusagenine (**7**) and flindersiamine (**3**) were more active against the human parasite *Trypanosoma cruzi* than the natural compounds without any cytotoxicity to three human cell types [41].

## 3. Materials and Methods

### 3.1. General

The fractionation process was conducted using a Biotage (Uppsala, Sweden) flash chromatography system equipped with a quaternary pump and a diode array detector set to 254 nm and 280 nm wavelengths. The fractions obtained were subjected to HPLC and TLC analysis. HPLC analysis was carried out using a 1260 Agilent HPLC system equipped with a Phenomenex Luna C18 column (250 × 4.6 mm; 10 μm). Alternatively, thin-layer chromatography plates (250 μm silica gel plates, Analtech, Newark, DE, USA) were employed, and a visual inspection was performed under UV light at 254 nm and 365 nm. Additionally, some fractions were visualized by spraying with anisaldehyde spray reagent or exposing them to I_2_ vapor.

A preparative HPLC system (Agilent 1200 Series, Santa Clara, CA, USA) was employed for final purifications. This system featured a G1361A binary pump, a G2260A autosampler, a G1315A diode array detector, and a G1364B fraction collector (Santa Clara, CA, USA). A Phenomenex Luna C_18_ column (250 × 21.2 mm; 10 μm) was used for this purpose.

In addition, 1D and 2D NMR spectra were recorded using a Bruker Avance III-400 MHz spectrometer, with CD_3_OD, CDCl_3_, or DMSO-*d*_6_ as solvents and TMS serving as the internal standard. Direct analysis of purified compounds in MeOH in real time–high-resolution mass spectrometry (DART-HRMS) was conducted using an AccuTOF-DART mass spectrometer (JEOL USA, Inc., Peabody, MA, USA).

### 3.2. Extraction of Plant Material

A sample of ground *H. parvifolia* stems was supplied by Dr. Charles Burandt at the University of Mississippi. Ground stems of *H. parvifolia* (450 g) were subjected to successive extractions with each solvent (2 L × 3) using an ultrasound sonicator bath. The extractions were carried out for 2 h at ambient temperature with both ethyl acetate and methanol. Subsequently, the solvents were evaporated under reduced pressure at 40 °C. This process yielded 8 g and 24 g of extracts from the ethyl acetate and methanol, respectively. These extracts were then evaluated for antifungal activity using bioautography and phytotoxicity using a seed germination assay. Only the ethyl acetate fraction exhibited positive activity.

### 3.3. Isolation of Phytotoxic Compounds

The ethyl acetate extract was fractionated using a 340 SNAP Biotage silica gel column using ethyl acetate in hexane (0−100%) gradient elution over 8 column volumes (582 mL each column volumes). The fractions were collected in 20 mL tubes and assessed with TLC. The fractions with similar phytochemical profiles were combined according to the TLC profile to obtain 20 fractions. These fractions were tested for phytotoxicity, and fractions 6, 8, and 14 had the highest phytotoxicity. They were then submitted to preparative HPLC to isolate their compounds. Fraction 6 was submitted to reversed-phase preparative HPLC with methanol (Solvent B) and water (Solvent A) in a gradient mode (30→40% of B in 4 min; 40→60% of B in 7 min; 60→80% of B in 10 min; hold 80% of B until 13 min; then 80→100% of B in 16 min) to give Compound **1** (rutacultin) as white crystals. High-resolution DART positive *m*/*z* 275.1325 [M + H]^+^, calculated for C_16_H_19_O_4_ 275.1283. ^1^H NMR (400 MHz, CDCl_3_) *δ* 7.53 (s, 1H, H-4), 6.87 (s, 1H, H-8), 6.83 (s, 1H, H-5), 6.37–6.04 (m, 1H, H-2′), 5.13 (s, 1H, H-3′), 5.09 (dd, *J* = 7.5, 1.0 Hz, 1H, H-3′), 3.94 (s, 3H, OCH_3_), 3.93 (s, 3H, OCH_3_), 1.50 (s, 6H, CH_3_). ^13^C NMR (100 MHz, CDCl_3_) *δ* 160.1 (C-2), 152.0 (C-7), 149.0 (C-9), 146.1 (C-6), 145.5 (C-2′), 137.6 (C-4), 132.1 (C-3), 112.1 (C-10), 111.7 (C-3′), 108.0 (C-5), 99.3 (C-8), 56.3 (OCH_3_), 56.3 (OCH_3_), 40.4 (C-1′), 26.0 (C-4′, C-5′) [42]. Compound **2** (pellitorine) as a white solid. High-resolution DART positive *m*/*z* 224.2007 [M + H]^+^, calculated for C_14_H_26_NO 224.2014. ^1^H NMR (400 MHz, CDCl_3_) *δ* 7.18 (dd, *J* = 15.0, 10.0 Hz, 1H, H-4), 6.18 (m, 1H, H-6), 5.75 (d, *J* = 15.0 Hz, 1H, H-5), 5.58 (t, *J* = 6.3 Hz, 1H, H-3), 3.15 (t, *J* = 6.5 Hz, 1H, H-1′), 2.13 (q, *J* = 6.9 Hz, 2H, H-7), 1.78 (h, *J* = 6.2 Hz, 1H, H-2′), 1.41 (p, *J* = 7.3 Hz, 2H, H-8), 1.29 (pt, *J* = 6.5, 2.5 Hz, 4H, H-9, H-10), 0.92 (s, 3H, H-3′), 0.91 (s, 3H, H-4′), 0.88 (t, *J* = 6.8 Hz, 3H, H-11). ^13^C NMR (100 MHz, CDCl_3_) *δ* 166.53 (C-2), 143.33 (C-4), 141.41 (C-6), 128.32 (C-5), 121.87 (C-3), 47.05 (C-1′), 33.05 (C-7), 31.50 (C-9), 28.76 (C-8), 28.62 (C-2′), 22.61 (C-10), 20.26 (C-3′, C-4′), 14.14 (C-11) [43]. Compound **3** (flindersiamine) as yellow powder. High-resolution DART positive *m*/*z* 274.0829 [M + H]^+^, calculated for C_14_H_12_NO_5_ 274.0715. ^1^H NMR (400 MHz, CDCl_3_) *δ* 7.59 (d, *J* = 2.8 Hz, 1H, H-2′), 7.28 (s, 1H, H-1′), 7.03 (d, *J* = 2.7 Hz, 1H, H-5), 6.07 (s, 2H, H-3′), 4.41 (s, 3H, OCH_3_), 4.28 (s, 3H, OCH_3_). ^13^C NMR (100 MHz, CDCl_3_) *δ* 162.62 (C-2), 156.08 (C-6), 146.74 (C-4), 143.03 (C-9), 138.03 (C-8), 137.74 (C-2′), 135.98 (C-7), 114.99 (C-10), 104.34 (C-1′), 102.93 (C-3), 101.52 (C-3′), 92.42 (C-5), 60.63 (OCH_3_), 58.96 (OCH_3_) [37].

Fraction 8 was subjected to reversed-phase HPLC preparative chromatography with a RP-C_18_ Phenomenex Luna C_18_ column (250 × 21.2 mm; 10 μm) using methanol (solvent B) and water (solvent A) in a gradient mode (40→60% of B in 3 min; 60→70% of B in 5 min; then 70→100% of B in 9 min) to obtain Compound **4** (chalepin) as a white solid. High-resolution DART positive *m*/*z* 315.1608 [M + H]^+^, calculated for C_19_H_23_O_4_ 315.1596. ^1^H NMR (400 MHz, CDCl_3_) *δ* 7.50 (s, 1H, H-5), 7.22 (s, 1H, H-4), 6.73 (s, 1H, H-8), 6.19 (m, 1H, H-2′), 5.15–5.05 (m, 2H, H-3′), 4.74 (t, *J* = 8.9 Hz, 1H, H-7′), 3.22 (ddd, *J* = 9.2, 7.3, 1.3 Hz, 2H, H-6′), 1.49 (s, 6H, CH_3_), 1.28 (s, 6H, CH_3_). ^13^C NMR (100 MHz, CDCl_3_) *δ* 162.23 (C-7), 160.18 (C-2), 154.65 (C-9), 145.62 (C-2′), 138.05 (C-4), 130.90 (C-3), 124.55 (C-6), 123.24 (C-5), 113.15 (C-10), 112.08 (C-3′), 97.16 (C-8), 94.03 (C-7′), 71.71 (C-8′), 40.31 (C-1′), 29.71 (C-6′), 26.12 (2CH_3_), 24.19 (2CH_3_) [44]. Compound **5** (1-hydroxy-3-methoxy-10-methylacridone) as yellow powder. High-resolution DART positive *m*/*z* 256.1063 [M + H]^+^, calculated for C_15_H_14_NO_3_ 256.0973. ^1^H NMR (400 MHz, CDCl_3_) *δ* 14.82 (s, 1H, OH), 8.43 (dd, *J* = 8.1, 1.7 Hz, 1H, H-5), 7.70 (ddd, *J* = 8.7, 7.0, 1.7 Hz, 1H, H-7), 7.46 (d, *J* = 8.7 Hz, 1H, H-8), 7.30–7.27 (m, 1H, H-6), 6.31–6.25 (m, 2H, H-1′, H-3′), 3.89 (s, 3H, OCH_3_), 3.77 (s, 3H, NCH_3_). ^13^C NMR (100 MHz, CDCl_3_) *δ* 180.76 (C-4), 166.03 (C-2′), 165.95 (C-4′), 144.70 (C-2), 142.38 (C-9), 134.05 (C-7), 126.73 (C-5), 121.41 (C-10), 121.03 (C-6), 114.49 (C-8), 105.26 (C-3), 94.05 (C-3′), 90.01 (C-1′), 55.58 (OCH_3_), 34.07 (NCH_3_) [45]. 

Fraction 14 was chromatographed using reversed-phase preparative HPLC with a gradient mode of methanol (solvent B) in water (solvent A) (50→60% of B in 3 min; 60→80% of B in 9 min; then 80→100% of B in 12 min) to yield Compound **6** (2-hydroxy-3,6,7-trimethoxyquinoline-4-carbaldehyde) as yellow powder. High-resolution DART positive *m*/*z* 264.0892 [M + H]^+^, calculated for C_13_H_14_NO_5_ 264.0872 (Supplementary Material, Figure S1). ^1^H NMR (400 MHz, DMSO-*d*_6_) *δ* 10.22 (s, 1H, 2′), 7.25 (s, 1H, H-5), 6.85 (s, 1H, H-8), 4.04 (s, 3H, OCH_3_), 3.85 (s, 3H, OCH_3_), 3.82 (s, 3H, OCH_3_) (Supplementary Material, Figure S2). ^13^C NMR (100 MHz, DMSO-*d*_6_) *δ* 189.94 (C-2′), 168.37 (C-2), 164.05 (C-3), 155.53 (C-6), 146.02 (C-7), 137.84 (C-9), 111.23 (C-10), 108.97 (C-4), 105.05 (C-8), 98.12 (C-5), 64.30 (OCH_3_), 56.36 (OCH_3_), 56.23 (OCH_3_) (Supplementary Material, Figure S3). Compound **7** (kokusagenine) as yellow crystals. High-resolution DART positive *m*/*z* 260.0992 [M + H]^+^, calculated for C_14_H_14_NO_4_ 260.0922. ^1^H NMR (400 MHz, CDCl_3_) *δ* 7.58 (d, *J* = 2.7 Hz, 1H, H-2′), 7.49 (s, 1H, H-5), 7.35 (s, 1H, H-8), 7.05 (d, *J* = 2.7 Hz, 1H, H-1′), 4.45 (s, 3H, OCH_3_), 4.04 (s, 3H, OCH_3_), 4.04 (s, 3H, OCH_3_). ^13^C NMR (100 MHz, CDCl_3_) *δ* 163.09 (C-2), 155.58 (C-4), 152.61 (C-7), 147.79 (C-6), 142.56 (C-9), 142.45 (C-2′), 112.96 (C-10), 106.70 (C-8), 104.62 (C-1′), 102.22 (C-3), 100.23 (C-5), 58.87 (OCH_3_), 56.05 (OCH_3_), 56.01 (OCH_3_) [37]. Compound **8** (maculine) as yellow crystals. High-resolution DART positive *m*/*z* 244.0598 [M + H]^+^, calculated for C_13_H_10_NO_4_ 244.0609. ^1^H NMR (400 MHz, CDCl_3_) *δ* 7.57 (d, *J* = 2.7 Hz, 1H, H-2′), 7.51 (s, 1H, H-5), 7.31 (s, 1H, H-8), 7.03 (d, *J* = 2.7 Hz, 1H, H-1′), 6.10 (s, 2H, H-3′), 4.41 (s, 3H, OCH_3_). ^13^C NMR (100 MHz, CDCl_3_) *δ* 163.14 (C-2), 155.98 (C-4), 150.76 (C-7), 146.09 (C-6), 143.85 (C-2′), 142.60 (C-9), 114.32 (C-10), 104.51 (C-8), 104.49 (C-1′), 102.50 (C-5), 101.60 (C-3), 98.03 (C-3′), 58.93 (OCH_3_) [37].

### 3.4. Bioassay for Phytotoxicity Evaluation with Lactuca sativa and Agrostis stolonifera

Plant extracts, fractions obtained through silica gel column chromatography, and isolated pure compounds were assessed for their phytotoxic effects on seeds of lettuce (*Lactuca sativa*) and bentgrass (*Agrostis stolonifera*), following the protocol described by Dayan et al. [46]. To assess phytotoxicity, seeds of *L. sativa* (Iceberg A Crisphead from Burpee Seeds) and *A. stolonifera* (Penncross variety, belonging to the creeping bentgrass species, sourced from Turf-Seed, Inc. in Hubbard, Oregon) were first subjected to surface sterilization by immersing them in a 2.5 % sodium hypochlorite for 10 min. Afterward, the seeds were thoroughly rinsed with sterile deionized water and air-dried in a sterile environment.

In a 24-well multiwell plate (Corning Inc., Corning, NY, USA), each well was filled with *A. solonifera* seeds (10 mg) or *L. sativa* (5 seeds) separately positioned on a filter paper (Whatman no. 1). The test compounds and fractions were dissolved in a mixture of acetone and DI water, ensuring a final acetone concentration of 10%. Subsequently, 200 μL of the test solution was added to each well containing seeds, while the control wells received only 200 μL of acetone and DI water. A 1 mM atrazine (ChemServices, West Chester, PA, USA) solution served as the positive control. The plate was covered and sealed using Parafilm and placed in a Percival Scientific CU-36L5 incubator, with continuous light conditions at 24 °C and an average photosynthetically active radiation (PAR) of 120 μmol s^−1^ m^−2^.

The phytotoxic activity was qualitatively assessed visually by comparing seed germination in each well after 7 days for *L. sativa* and after 10 days for *A. stolonifera*, using a rating scale ranging from 0 to 5. A rating of 0 indicated no effect (all seeds germinated), while a rating of 5 indicated no seed germination [46]. Each experiment was conducted in triplicate.

### 3.5. Phytotoxicity Assay with Lemna paucicostata 

*Lemna paucicostata* cultures were cultivated from a single colony, comprising a mother and two daughter fronds, in a beaker filled with Hoagland’s No. 2 Basal Salt Mixture (Sigma H2395, San Luis Obispo, CA, USA) at a concentration of 1.6 g/L, supplemented with iron (1 mL of 1000× FeEDTA solution per 1 L of Hoagland media). The pH of the medium was adjusted to 5.5 using 1 N NaOH and then filter-sterilized through a 0.2 μm filter. These *L. paucicostata* cultures were grown in approximately 100 mL of sterile jars with vented lids in a Percival Scientific CU-36L5 incubator, maintaining continuous light conditions at 24 °C and an average 120 μmol s^−1^ m^−2^ PAR. The doubling time for the plants was approximately 24 to 36 h. Nonpyrogenic polystyrene sterile six-well plates (CoStar 3506, Corning Incorporated, Wilmington, NC, USA) were used for assays. Each well contained 4950 μL of Hoagland’s media and 50 μL of water, solvent, or the compound dissolved in the appropriate solvent, resulting in a final solvent concentration of 1% by volume. Atrazine was used as the positive control. Two three-frond plants of the same age (4 to 5 days old) and approximate size were inoculated into each well. As mentioned earlier, all six-well plates were placed in the Percival incubator, maintaining conditions at 24 °C and a 120 μmol s^−1^ m^−2^ average PAR. The LabScanalyzer (LemnaTec Gmbh, Aachen, GER), an image analyzer was used to measure the frond surface area. The measurements were recorded on day 0 and day 7. The dose-response analysis and calculation of half-maximal inhibitory concentration (IC_50_) was performed with R 4.2.1 software with support of the drc package. 

### 3.6. Antifungal Bioautography Assay

The assessment of antifungal activity against fungal plant pathogens followed a published TLC bioautography method [47]. We selected a fungal crop pathogen *Colletotrichum fragariae* (isolate cf63) that infects strawberries and many other vegetables and fruits. Pure compounds (10 μL) were dissolved in methanol and then applied at concentrations of 10, 20, 50, and 100 μM into silica gel TLC plates (250 μm, silica gel GF Uniplate; Analtech, Inc., Jalan Pemimpin, Singapore). After solvent evaporation, these plates were then sprayed with spore suspensions of *C. fragariae*, adjusted to a final concentration of 3.0 × 10^5^ conidia/mL using potato dextrose broth (PDB, Difco, Sparks, MD, USA) and 0.1% Tween-80. The TLC plate was sprayed until it appeared damp with the prepared conidial suspension (approx. 1 mL/plate). The inoculated TLC plates, placed in moisture chamber (30 × 13 × 7.5 cm) boxes to maintain a 100% relative humidity, were incubated in a growth chamber for 4 days, maintained at 27 ± 1 °C, with a 12 h photoperiod under photon flux conditions of 60 ± 5 μmol s·m^−2^ s^−1^. To determine the sensitivity of each tested compound against fungal species, inhibitory zone areas were compared. Bioautography experiments were conducted in triplicate, including both dose-response and non-dose-response assessments. A technical grade fungicide standard, captan (98%; Chem Service, Inc., West Chester, PA, USA), served as the positive control.

## Figures and Tables

**Table 1:** Phytotoxicity of atrazine and compounds isolated from stem ethyl acetate extract of *Helietta parvifolia*. All compounds were tested at 1 mM.

Compounds	Ranking	
	*L. sativa*	*A. stolonifera*
**1**	1	1
**2**	0	2
**3**	0	2
**4**	0	3
**5**	0	0
**6**	0	3
**7**	0	0
**8**	0	0
atrazine	3	4

Ranking based on scale of 0 to 5; 0 = no effect; 5 = no germination.

## Data Availability

Data are contained within the article and Supplementary Materials.

## Supplementary Materials

The following supporting information can be downloaded at https://www.mdpi.com/article/10.3390/molecules28237930/s1, Figure S1: HR-DART-MS of compound **6** in positive mode; Figure S2: ^1^H NMR spectrum of compound **6** in DMSO-*d*_6_ (400 MHz); Figure S3: ^13^C NMR spectrum of compound **6** in DMSO-*d*_6_ (100 MHz); Figure S4: HMBC NMR spectrum of compound **6** in DMSO-*d*_6_; Figure S5: HSQC NMR spectrum of compound **6** in DMSO-*d*_6_.
