# Supplementary material for: Antifungal and Phytotoxic Activities of Isolated Compounds from Helietta parvifolia Stems
Source: Molecules. 2023 Dec 4;28(23):7930. doi: 10.3390/molecules28237930 (PMC10708216; doi:10.3390/molecules28237930)
Supplement: Supplementary file 1 [file molecules-28-07930-s001.zip › molecules-2674863-supplementary.pdf]

Supplementary material

Antifungal and Phytotoxic activities of isolated compounds from *Helietta parvifolia* stems.

Victor Pena Ribeiro[1,2], Joanna Bajsa-Hirschel[2], Prabin Tamang[2], Kumudini Meepagala[2*] Stephen O. Duke[3]

[1]ORISE Fellow-Agricultural Research Service, U.S. Department of Agriculture, Natural Products Utilization Research Unit, P.O. Box 1848, University, MS, 38677, USA

[2]Agricultural Research Service, U.S. Department of Agriculture, Natural Products Utilization Research Unit, P.O. Box 1848, University, MS, 38677, USA

[3]National center for Natural products Research, University, MS 38677, USA

Abstract: The identification of natural and environmentally friendly pesticides is a key area of interest for the agrochemical industry, with many potentially active compounds being sourced from numerous plant species. In this study, we report the bioassay-guided isolation and identification of phytotoxic and antifungal compounds from the ethyl acetate extract of *Helietta parvifolia* stems. We identified eight compounds, consisting of two coumarins and six alkaloids. Among these, a new alkaloid, 2-hydroxy-3,6,7-trimethoxyquinoline-4-carbaldehyde (6), was elucidated, along with seven known compounds. The phytotoxicity of purified compounds was evaluated, and chalepin (4) was active against *Agrostis stolonifera* at 1 mM with 50% inhibition of seed germination and reduced *Lemna pausicotata* (duckweed) growth by 50% (IC50) at 168 μM. Additionally, we evaluated the antifungal activity against the fungal plant pathogen *Colletotrichum fragariae* using a thin-layer chromatography bioautography assay, which revealed that three isolated furoquinoline alkaloids (flindersiamine (3), kokusagenine (7), maculine (8) among the isolated compounds, had the strongest inhibitory effects on the growth of *C. fragariae* at all tested concentrations. Our results indicate that these active natural compounds (3), (4), (7), and (8) could be scaffolds for production of more active pesticides with better physicochemical properties.

Keywords: *Helietta parvifolia*, herbicidal, fungicidal.

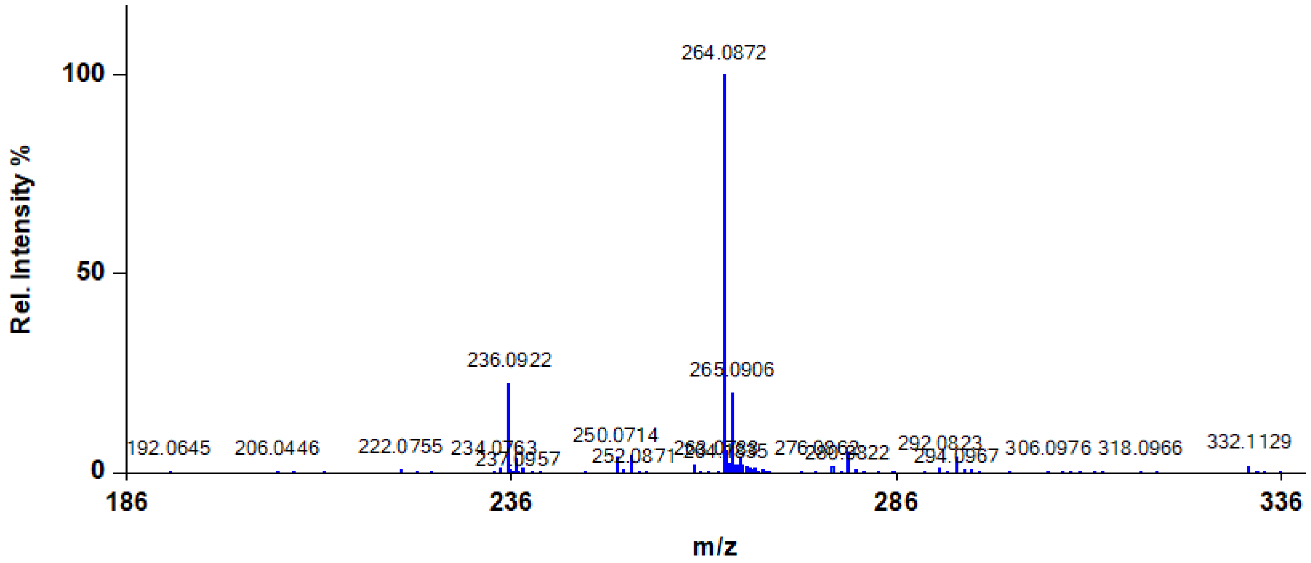

Elemental Compositions

Element Limits: C 0/50 H 0/100 O 0/10 N 0/10

Tolerance: 2 mmu   Even or odd electron ion or both: Both

Electron correction: None. Charges: 1

Minimum unsaturation: -1   Maximum unsaturation: 100

Calc. m/z	Abund %	mmu	Peaks	Score	DBE	Composition	NIST
264.087197	0.61	0.04	3	0.000770	7.5	C13H14O5N1	0
264.087190	0.46	0.14	3	0.002216	13.0	C12H8N8	0
264.088533	0.33	0.74	3	0.008270	12.5	C14H10O1N5	0
264.085854	0.64	0.73	4	0.011604	8.0	C11H12O4N4	0
264.089040	1.84	1.12	3	0.068776	0.0	H12O7N10	0

Figure S1. HR-DART-MS of compound **6** in positive mode

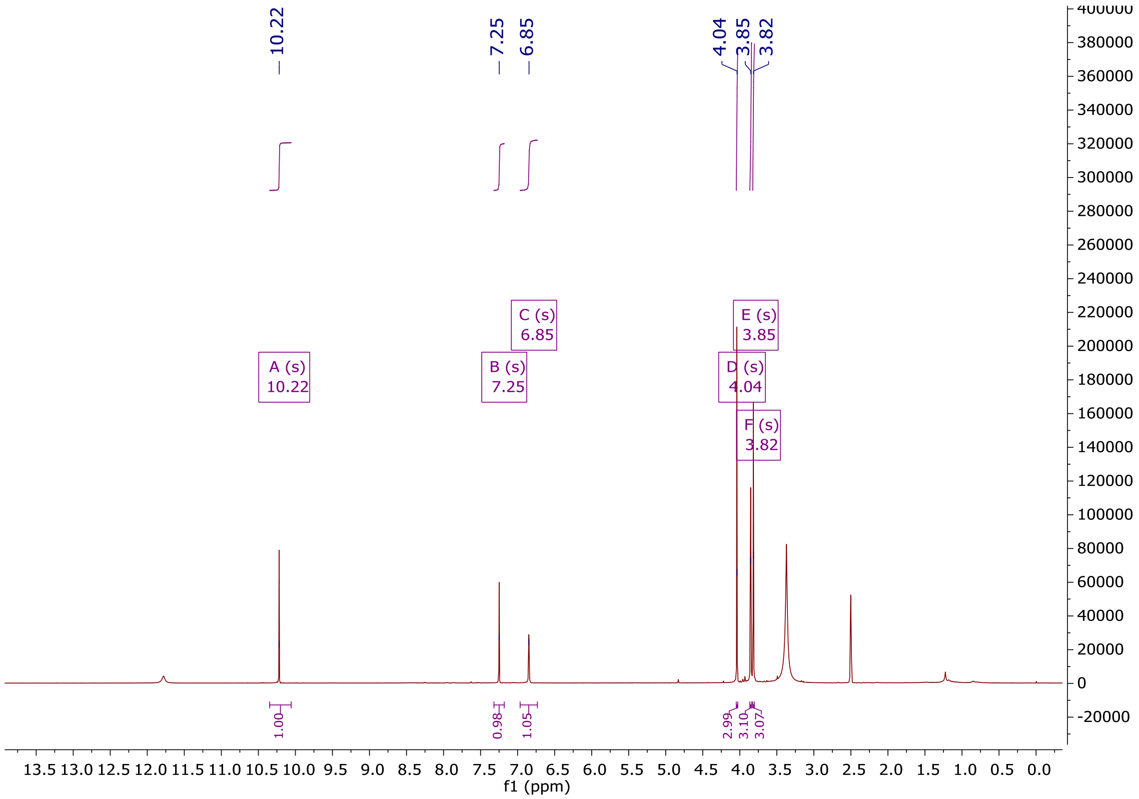

Figure S2. ^{1}H NMR spectrum of compound **6** in DMSO-d6 (400 MHz).

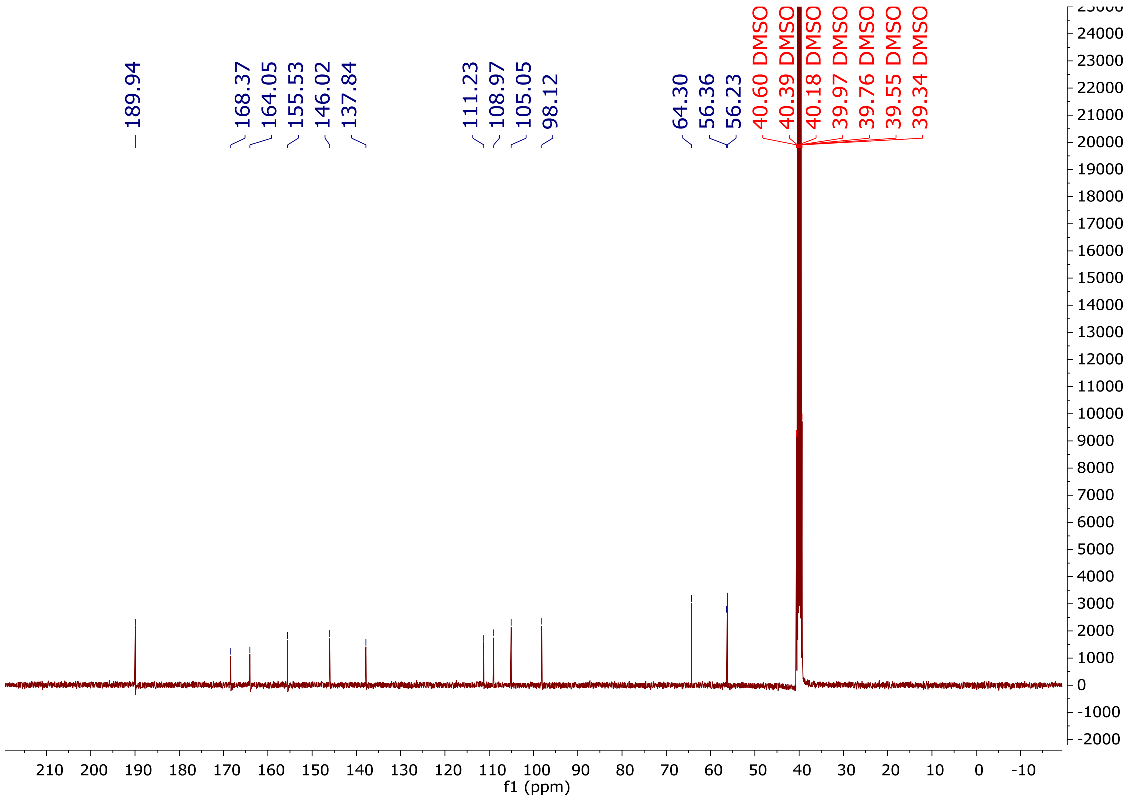

Figure S3. ^{13}C NMR spectrum of compound **6** in DMSO-d6 (100 MHz).

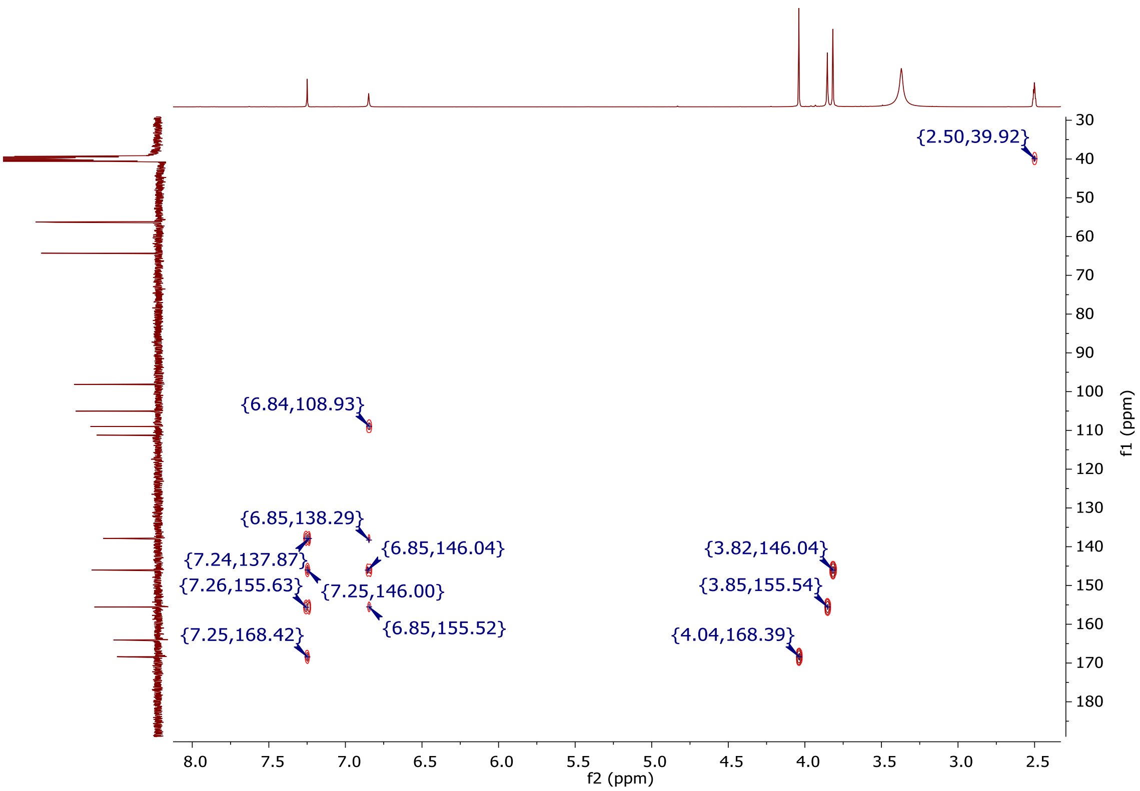

Figure S4. HMBC NMR spectrum of compound **6** in DMSO-d6

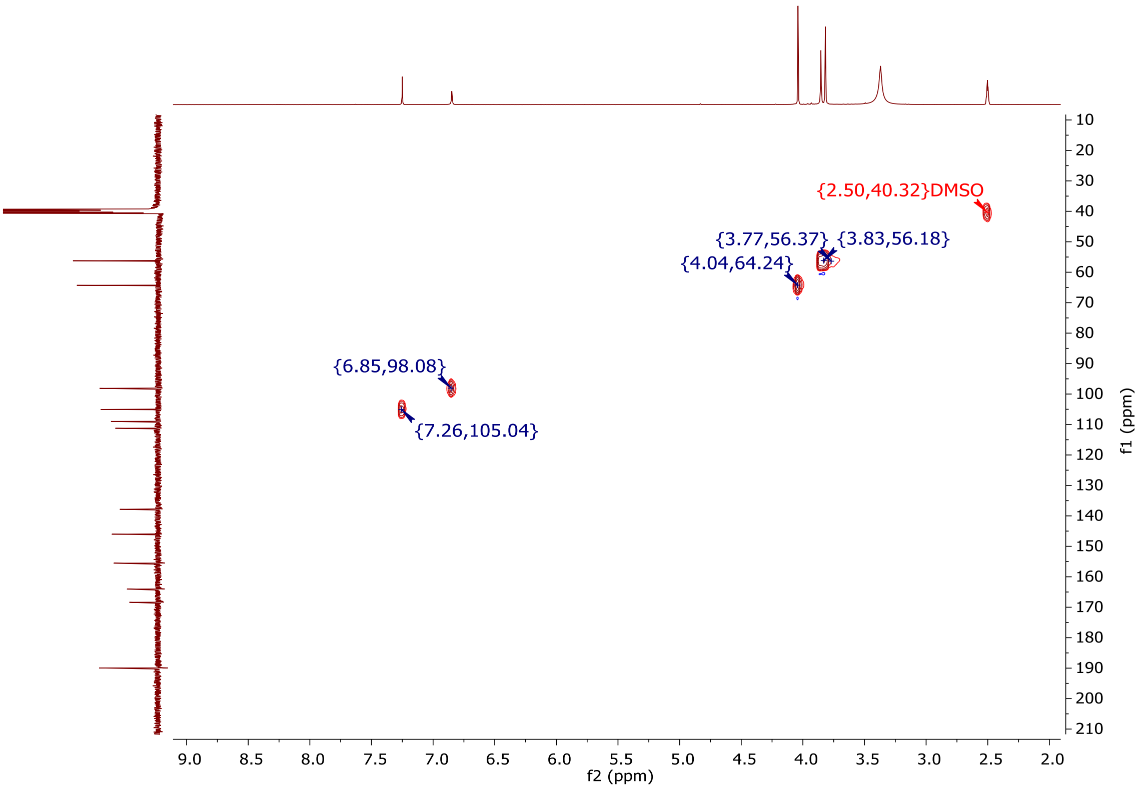

Figure S5. HSQC NMR spectrum of compound **6** in DMSO-d6
